# Type 3 inositol 1,4,5-trisphosphate receptor is dispensable for sensory activation of the mammalian vomeronasal organ

**DOI:** 10.1038/s41598-017-09638-8

**Published:** 2017-08-31

**Authors:** Pablo Chamero, Jan Weiss, María Teresa Alonso, Macarena Rodríguez-Prados, Chihiro Hisatsune, Katsuhiko Mikoshiba, Trese Leinders-Zufall, Frank Zufall

**Affiliations:** 1 Center for Integrative Physiology and Molecular Medicine, Saarland University, 66421 Homburg, Germany; 2 Laboratoire de Physiologie de la Reproduction et des Comportements, UMR 0085 INRA-CNRS-IFCE-Université de Tours, Nouzilly, 37380 France; 3 Instituto de Biología y Genética Molecular (IBGM), University of Valladolid and CSIC, 47003 Valladolid, Spain; 4 Laboratory for Developmental Neurobiology, RIKEN Brain Science Institute, Saitama, 351-0198 Japan

## Abstract

Signal transduction in sensory neurons of the mammalian vomeronasal organ (VNO) involves the opening of the canonical transient receptor potential channel Trpc2, a Ca^2+^-permeable cation channel that is activated by diacylglycerol and inhibited by Ca^2+^-calmodulin. There has been a long-standing debate about the extent to which the second messenger inositol 1,4,5-trisphosphate (InsP_3_) and type 3 InsP_3_ receptor (InsP_3_R3) are involved in the opening of Trpc2 channels and in sensory activation of the VNO. To address this question, we investigated VNO function of mice carrying a knockout mutation in the *Itpr3* locus causing a loss of InsP_3_R3. We established a new method to monitor Ca^2+^ in the endoplasmic reticulum of vomeronasal sensory neurons (VSNs) by employing the GFP-aequorin protein sensor erGAP2. We also performed simultaneous InsP_3_ photorelease and Ca^2+^ monitoring experiments, and analysed Ca^2+^ dynamics, sensory currents, and action potential or field potential responses in InsP_3_R3-deficient VSNs. Disruption of *Itpr3* abolished or minimized the Ca^2+^ transients evoked by photoactivated InsP_3_, but there was virtually no effect on sensory activation of VSNs. Therefore, InsP_3_R3 is dispensable for primary chemoelectrical transduction in mouse VNO. We conclude that InsP_3_R3 is not required for gating of Trpc2 in VSNs.

## Introduction

The mammalian olfactory system has evolved two major signaling systems for chemoelectrical transduction: one that depends on cyclic nucleotide-gated (CNG) channel activation and involving cAMP or cGMP signaling, respectively, and another that depends on transient receptor potential (TRP) channel activation, mainly involving the Trpc2 cation channel^1, 2^. Trpc2 is a central transduction element in sensory neurons of the mouse vomeronasal organ (VNO)^3–5^ which play important roles in the detection of socially-relevant molecular cues such as pheromones and kairomones^6–8^. The recent finding that Trpc2 is also expressed in sensory neurons of the main olfactory epithelium (MOE)^9, 10^ where it is required in type B cells for the detection of low environmental oxygen^11^ has sparked renewed interest in its function. Despite two decades of research, the second messenger signaling mechanisms underlying activation of Trpc2 and its corresponding sensory responses are still debated and there is presently no single agreed-upon mechanism for its activation^7, 12–15^. Moreover, the behavioral phenotypes of *Trpc2* mutant mice are more complex than previously thought^16^. A complete understanding of Trpc2 signaling mechanisms will be required to fully appreciate the role of Trpc2 in chemical communication and social behaviors.

Here we assess the impact of the second messenger inositol 1,4,5-trisphosphate (InsP_3_) and the type 3 InsP_3_ receptor (InsP_3_R3, encoded by the gene *Itpr3*) on chemodetection and transduction of mouse vomeronasal sensory neurons (VSNs), and thus on the activation of Trpc2 channels. VSN chemodetection depends on several large G-protein-coupled receptor families (GPCRs), predominantly the vomeronasal type 1 and type 2 receptors, whose activation stimulates phospholipase C (PLC) leading to the hydrolysis of phosphatidylinositol-4,5-bisphosphate (PIP_2_) into InsP_3_ and diacylglycerol (DAG)^2, 6, 17, 18^. Trpc2 was initially proposed to function as a Ca^2+^ store-activated, capacitative Ca^2+^ entry (CRAC) channel^19–21^ and several studies suggested a critical role for InsP_3_ in VSN chemotransduction^22–27^. More recently, a transduction model has been presented in which InsP_3_R3 is physically linked to Trpc2 and thereby would directly contribute to its activation^28, 29^. However, because Trpc2 channels are highly localized in VSN microvilli, at a considerable distance from Ca^2+^ stores, the validity of such a model has been questioned^12, 30, 31^. Our own studies concluded that Trpc2 is a DAG-activated, Ca^2+^-permeable cation channel that can be inhibited by Ca^2+^-calmodulin (Ca^2+^-CaM) and does neither require InsP_3_ nor Ca^2+^ stores for its activation in VSNs^5, 7, 18, 32^. Since then, there has been considerable evidence for the existence of additional, sequential or parallel VSN signaling mechanisms some of which require elevated intracellular Ca^2+^ levels and could thus depend on InsP_3_R3 activation and store-dependent Ca^2+^ mobilization^16^. These mechanisms include the activity of Ca^2+^-activated chloride channels^33–37^, several types of Ca^2+^-activated potassium channels^38^, as well as Ca^2+^-activated cation channels^32, 39^.

To resolve these problems and to further define the nature of the signal transduction mechanism in VSNs, we investigated VNO function in a gene-targeted mouse strain that carries a knockout mutation in the *Itpr3* locus and thus lacks InsP_3_R3^40, 41^. We used a recently developed method to monitor Ca^2+^ in the endoplasmic reticulum (ER) by employing a novel, genetically-encoded Ca^2+^ sensor protein specifically targeted to the ER (erGAP2)^42, 43^, and we performed simultaneous InsP_3_ photorelease and Ca^2+^ monitoring experiments. By applying a combination of state-of-the-art Ca^2+^ imaging and electrophysiological methods using wild-type and InsP_3_R3-deficient VSNs, we asked the following: (1) Are intracellular Ca^2+^ stores significantly mobilized during VSN sensory activation? (2) Does InsP_3_ induce Ca^2+^ signals in VSNs? (3) Is InsP_3_R3 necessary for stimulus-evoked Ca^2+^ signaling, the generation of sensory currents, and action potential or field potential responses in VSNs? With this approach, we provide compelling evidence that sensory activation of the mouse VNO is largely independent of InsP_3_R3, ruling out a crucial role for InsP_3_ signaling in the primary chemotransduction process of mouse VSNs.

## Results

### Monitoring [Ca^2+^]_ER_ after VSN stimulation

**Figure 1:**
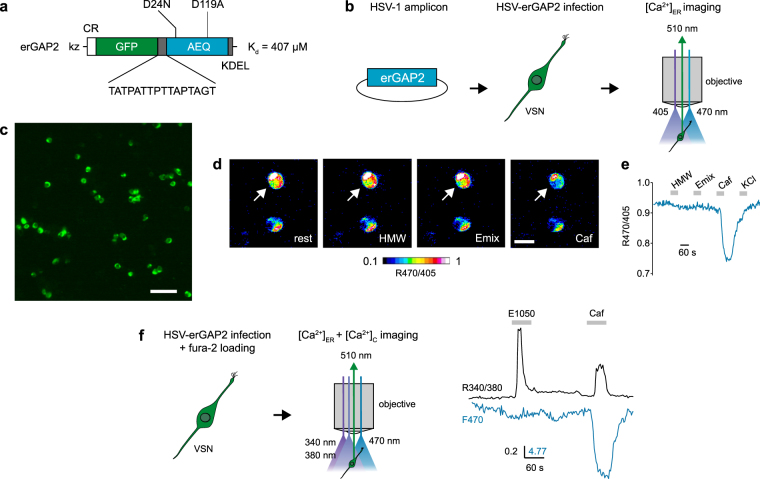
erGAP2 Ca^2+^ reporter enables imaging of [Ca^2+^]_ER_ in VSNs. (**a**) Domain structure of the erGAP2 construct. Kz, Kozac sequence; CR, calreticulin signal sequence; GFP, green fluorescent protein; AEQ, aequorin; KDEL, ER retention signal. (**b**) erGAP2 is inserted into a p-HSV-1 amplicon expression vector, and HSV-1 viruses are prepared and used to infect freshly dissociated VSNs for 24 h. Ratiometric imaging is performed by double excitation at 405/470 nm and single emission at 510 nm. (**c**) Fluorescent image (470 nm excitation) showing expression of erGAP2 in VSNs. Freshly dissociated cells were infected with HSV-erGAP2 for 24 h. Scale bar, 50 μm. (**d**) erGAP2 ratiometric imaging in single VSNs. Stimulation with caffeine (Caf, 50 mM) but not with HMW or E mix caused a reduction in the F_470/405_ ratio. Scale bar, 10 μm. (**e**) Analysis of F_470/405_ ratio over time from the cell shown in **d** (arrow). (**f**) Simultaneous ER and cytosolic [Ca^2+^] recordings on VSNs infected with HSV-erGAP2 and loaded with fura-2. Imaging is performed by triple excitation with 340/380/470 nm light and single emission at 510 nm. [Ca^2+^]_C_ is estimated by 340/380 ratio and [Ca^2+^]_ER_ by 470 nm single excitation. Example of a cell showing cytosolic Ca^2+^ transients to the sulfated steroid E1050 (100 μM) and caffeine (50 mM), and a simultaneous decrease in [Ca^2+^]_ER_ only in response to caffeine.

To investigate the role of InsP_3_ receptors (InsP_3_Rs) and intracellular Ca^2+^ stores in stimulus-evoked responses of mouse VSNs, we first monitored Ca^2+^ signals in the ER lumen of isolated VSNs. The ER is the main Ca^2+^-storage organelle of the cell, as the Ca^2+^ concentration in ER lumen ([Ca^2+^]_ER_) is commonly 10^4^-fold higher than cytosolic Ca^2+^ ([Ca^2+^]_C_). Conventional Ca^2+^ indicators pose serious limitations for monitoring [Ca^2+^]_ER_ in terms of Ca^2+^ affinity, organelle selectivity and/or dynamic range. To overcome these difficulties, we used a novel low-Ca^2+^ affinity sensor, erGAP2, that can be targeted specifically to the ER lumen^42^. erGAP2 is a fluorescent, genetically-encoded Ca^2+^ indicator based on the fusion of two jellyfish proteins, GFP and aequorin. erGAP2 has been optimized for measurements in high Ca^2+^ concentration environments (K_d_ for Ca^2+^  = 407 µM) due to two substitutions on the EF hands of the Ca^2+^-binding domain (Fig. 1a). Targeting to the ER is achieved through a combination of the calreticulin signal peptide and the ER retention sequence KDEL flanking the sensor (Fig. 1a). This reporter enables ratiometric imaging using two excitation peaks at 405 and 470 nm and single emission at 510 nm (Fig. 1b)^43^.

We expressed erGAP2 in freshly dissociated VSNs from C57BL/6 (wild-type, WT) mice using a herpes simplex virus type 1 (HSV-1) amplicon vector^44^ (Fig. 1b). A 24-h incubation period was sufficient to observe robust erGAP2 expression in infected VSNs with intact cell viability (Fig. 1c). We performed ratiometric [Ca^2+^]_ER_ imaging on infected VSNs and analysed stimulus-induced fluorescence signals (Fig. 1d). We compared response patterns to several previously established VSN chemostimuli: a mix of sulfated steroids (E1100, E0893, E0588, and E1050, each at 100 µM), and the high molecular weight fraction of mouse urine (HMW; 1:300) which contains major urinary proteins (MUPs). These stimuli have been reported to activate VSNs of both the apical (sulfated steroids) and basal (HMW) layers of the VNO neuroepithelium^45–49^ and their responses are known to depend on Trpc2^45, 46^. We also applied caffeine (Caf, 50 mM), a potent activator of the ubiquitous ryanodine receptors^50^, to induce Ca^2+^ release from the ER. We measured F_470_/F_405_ ratios in 368 cells that expressed detectable erGAP2 fluorescence. All cells showed a decrease of the F_470_/F_405_ ratio in response to a 60-s caffeine stimulus, indicative of a sharp decrease of [Ca^2+^]_ER_. In contrast, stimulation with the mix of sulfated steroids (E mix) or HMW did not induce major changes in F_470_/F_405_ ratio. Imaging of a representative cell and its F_470_/F_405_ ratio trace as a function of time is displayed in Fig. 1d and e, respectively. On average, both E mix and HMW induced no or only very minor reductions in F_470_/F_405_ ratio (−0.0117 ± 0.0033 and −0.0155 ± 0.0035, respectively) whereas caffeine produced a robust, 15-fold larger decrease in F_470_/F_405_ ratio (−0.175 ± 0.0071; Mann-Whitney test, n = 368, P = 1.6 × 10^−85^ and 5.6 × 10^−83^).

### Simultaneous [Ca^2+^]_ER_ and [Ca^2+^]_C_ imaging in VSNs

To determine whether VSNs activated by HMW or steroid ligands show Ca^2+^ release from the ER, we performed simultaneous measurements of [Ca^2+^]_ER_ and [Ca^2+^]_C_ using combined imaging of erGAP2 and the cytosolic Ca^2+^ dye fura-2. For this approach, we imaged erGAP2-expressing cells loaded with fura-2 and performed triple illumination with 340 nm, 380 nm and 470 nm excitation light and single channel emission at 510 nm (Fig. 1f). Changes in [Ca^2+^]_C_ were estimated by calculating the 340/380 nm ratio (R_340/380_), and [Ca^2+^]_ER_ was estimated by using single F_470_ dynamics because of the partial F_405_ overlap with fura-2. We imaged a total of 599 erGAP2-expressing cells and applied 60-s stimuli of either caffeine, HMW, or the sulfated steroid estradiol-3,17-disulfate (E1050; 100 µM). We observed robust [Ca^2+^]_C_ increases, measured as a rise in R_340/380_, in 15 cells responding to HMW and in 8 cells responding to E1050. These cells also showed [Ca^2+^]_C_ increases in response to caffeine that were accompanied by simultaneous reductions in F_470_, indicative of massive Ca^2+^ release from the ER (Fig. 1f).

By contrast, changes in F_470_ in response to E1050 and HMW were nearly absent or not existent in these cells (Fig. 1f and below), indicating that [Ca^2+^]_ER_ remains largely unaffected during activation with E1050 and HMW. On average, caffeine induced a 10-fold larger reduction of F_470_ (−3.6 ± 0.4848 a.u.; n = 23) than HMW (−0.3 ± 0.1393 a.u.; Mann-Whitney test, P = 7.1 × 10^−7^; n = 15) and E1050 (Mann-Whitney test, P = 7.7 × 10^−7^; n = 8). E1050 even induced a small F_470_ increase of 0.07 ± 0.3305 a.u., not significantly different from HMW (Mann-Whitney test, P = 0.38). Importantly, average caffeine-induced [Ca^2+^]_C_ peaks were similar to those induced by HMW (0.4 ± 0.0982 vs. 0.56 ± 0.1288, respectively; Mann-Whitney test, P = 0.14) or even smaller than E1050 peaks (0.75 ± 01734; Mann-Whitney test, P = 0.04). Therefore, the main Ca^2+^ source contributing to HMW- and E1050-induced elevations of [Ca^2+^]_C_ does not originate from ER stores.

### Removal of extracellular Ca^2+^ abolishes ligand-induced VSN responses

**Figure 2:**
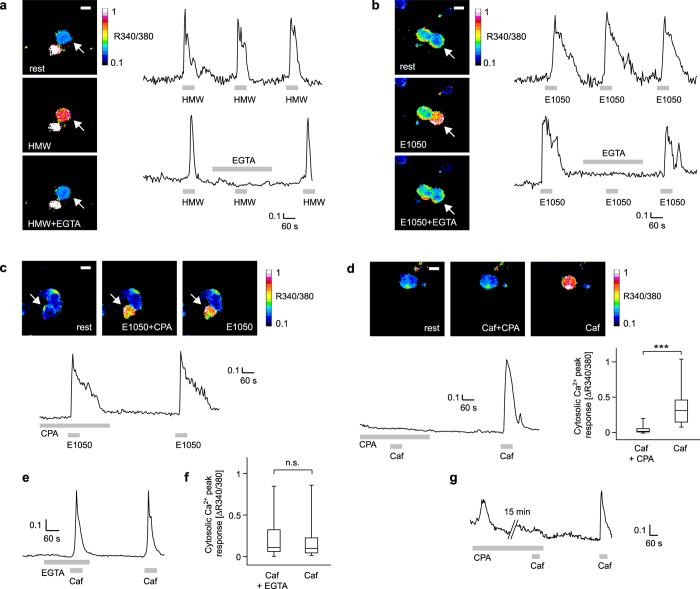
Fura-2 Ca^2+^ imaging in freshly dissociated VSNs. Examples of cells imaged with fura-2 (R_340/380_ ratio images) and corresponding time courses showing cytosolic Ca^2+^ transients evoked by urine HMW fraction (1:300 dilution) (**a**) and E1050 (**b**). Cytosolic Ca^2+^ transients to both types of stimuli are abolished in the absence of extracellular Ca^2+^ (EGTA 0.5 mM), an effect that is reversible. (**c**) Depletion of [Ca^2+^]_ER_ by a 20-min pre-incubation (only last 5 min shown) with the SERCA blocker CPA (30 µM) has no effect on E1050-induced cytosolic Ca^2+^ transients. (**d**) CPA treatment abolishes caffeine-evoked cytosolic Ca^2+^ signals. Recording example and group data showing that responses to 50 mM caffeine (Caf) are nearly abolished after a 20-min CPA (30 µM) incubation (only last 5 min shown), with a recovery after a 5-min washout (n = 14; Wilcoxon signed-rank test: P < 0.001). (**e**) Recording example and (**f**) group data of caffeine stimulation during and after incubation with or without 0.5 mM EGTA, showing that [Ca^2+^]_ER_ is not depleted (n = 39; Wilcoxon signed-rank test, P = 0.166). (**g**) Recording example showing a CPA-mediated intracellular Ca^2+^ rise at the beginning of a 20-min incubation period (experimental design as in (**d**); n = 14). Scale bars, 10 µm. Median values and interquartile ranges are shown in box plots.

To assess whether extracellular Ca^2+^ is necessary to generate elevations of [Ca^2+^]_C_ following stimulation with HMW and E1050, we performed fura-2 imaging in freshly dissociated, non-infected VSNs^45, 47, 51^. We first established that multiple, 60-s stimulations with HMW or E1050 that were separated by 4-min interstimulus intervals are sufficient to produce robust and repeatable increases in [Ca^2+^]_C_ (Fig. 2a and b). Next, we used a protocol in which a Ca^2+^-free extracellular medium containing 0.5 mM EGTA was applied during the second stimulation with either HMW or E1050. Ca^2+^ responses were completely absent during the second stimulus application in Ca^2+^-free medium for all cells that responded to any of the two stimuli during the first application (HMW, n = 3; E1050, n = 8; Fig. 2a and b). When extracellular Ca^2+^ was re-introduced, [Ca^2+^]_C_ increases recovered during a third application (Fig. 2a and b). To verify that Ca^2+^ stores were not depleted under these conditions, we applied caffeine in Ca^2+^-free medium and observed robust Ca^2+^ transients (Fig. 2e). These responses were very similar to caffeine responses obtained in Ca^2+^-containing medium after a 4-min recovery period (Fig. 2e and f; Wilcoxon signed-rank test, P = 0.166) indicating that internal Ca^2+^ stores were not depleted. Therefore, extracellular Ca^2+^ and Ca^2+^ entry is required to generate cytosolic Ca^2+^ transients in response to activation with HMW and E1050, consistent with previous results^47, 52^.

### Depletion of intracellular Ca^2+^ stores does not affect ligand-induced Ca^2+^ responses

To further explore the impact of intracellular Ca^2+^ stores on stimulus-evoked VSN responses, we applied cyclopiazonic acid (CPA), a potent inhibitor of sarcoplasmic-endoplasmic reticulum Ca^2+^-ATPases (SERCAs), in order to deplete intracellular Ca^2+^ stores. VSNs were incubated with 30 µM CPA for 20 min in an extracellular medium containing Ca^2+^. We then performed fura-2 imaging during stimulation with HMW or E1050 in the presence of CPA. We observed robust increases of [Ca^2+^]_C_ in 4/140 cells stimulated with HMW and in 7/250 cells stimulated with E1050 (Fig. 2c). Unlike other SERCA inhibitors such as thapsigargin, the effect of CPA can be reversed after washout. We used 60-s caffeine pulses (50 mM) to determine that a 5-min washing period after CPA incubation was sufficient to restore [Ca^2+^]_ER_ and enable robust Ca^2+^ release (Fig. 2d). Caffeine-induced Ca^2+^ responses increased drastically after CPA washout (Wilcoxon signed-rank test, P < 0.001; Fig. 2d). We compared the cell-to-cell peak amplitudes of HMW and E1050 during CPA treatment (0.2788 ± 0.0579, HMW; 0.5153 ± 0.0745, E1050) vs. washout (0.3018 ± 0.0766, HMW; 0.4058 ± 0.0611, E1050). Although E1050 responses tended to be slightly larger in the presence of CPA, we found no significant differences between the two conditions (Wilcoxon signed-rank test, P = 0.58, HMW; P = 0.07, E1050). To determine the effect of CPA application on intracellular Ca^2+^, we recorded the first 5 min after CPA incubation and observed a slow and transient Ca^2+^ increase (n = 14; Fig. 2g), consistent with sustained, store-dependent Ca^2+^ release.

Together, these results indicate that depletion of intracellular Ca^2+^ stores does not prevent the generation of cytosolic Ca^2+^ transients following activation with HMW and E1050. Thus, intracellular Ca^2+^ stores are not critical for VSN cytosolic Ca^2+^ transients to these sensory stimuli and extracellular Ca^2+^ must be the main source of cytosolic Ca^2+^ for these Ca^2+^ responses.

### InsP_3_R3 is expressed in mouse VSNs

**Figure 3:**
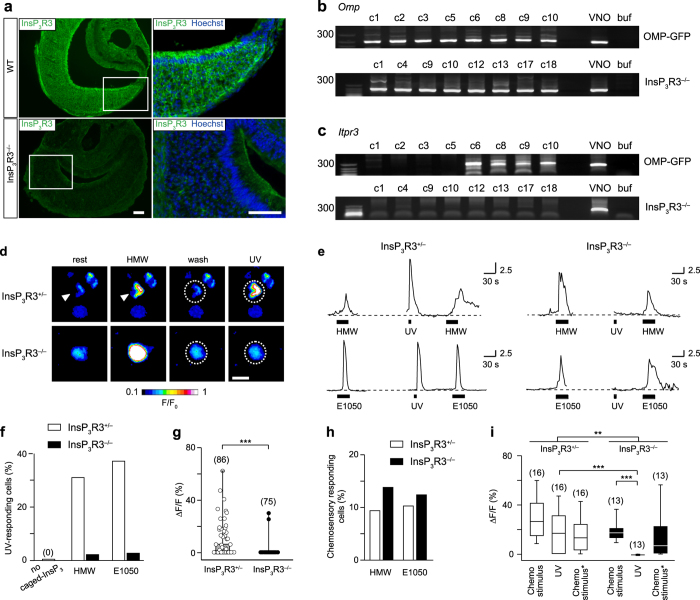
InsP_3_R3 is expressed in VSNs and is required for Ca^2+^ responses to photoreleased InsP_3_. (**a**) Immunolabeling of VNO sections with anti-InsP_3_R3 antibody (green) and the nuclear dye Hoechst 33342 (blue) in VNOs from WT and InsP_3_R3^−/−^ mice. Scale bars, 100 µm. (**b**) Single-cell RT-PCR for olfactory marker protein (*Omp*) and (**c**) *Itpr3* genes in single VSNs from OMP-GFP or InsP_3_R3^−/−^ mice. Bands positive for *Omp* were amplified in 8/10 GFP + cells (OMP-GFP) and in 8/18 non-labeled cells (InsP_3_R3^−/−^). From these *Omp* + cells, *Itpr3* RT-PCR product was amplified in 4 cells but in none of the InsP_3_R3^−/−^ cells. Full-length gels are presented in Supplementary Fig. S1. (**d**) Photolysis of caged InsP_3_ in isolated VSNs. Fluo-4 confocal images of VSNs loaded with caged InsP_3_. Both InsP_3_R3^+/−^ (top, arrowhead) and InsP_3_R3^−/−^ (bottom) cells showed responses to HMW. Outlined regions of cells activated by HMW (dotted areas) were stimulated with UV light causing a Ca^2+^ increase in the InsP_3_R3^+/−^ but not in the InsP_3_R3^−/−^ cell. Scale bar, 10 µm. (**e**) Examples of Ca^2+^ responses in InsP_3_R3^+/−^ (left) and InsP_3_R3^−/−^ (right) cells stimulated with HMW fraction (1:100), E1050 (100 µM) and UV light. (**f**) Photoactivation efficiency of InsP_3_R3^+/−^ cells loaded with caged InsP_3_ was between 30 and 40%. (**g**) Comparison of Ca^2+^ peak amplitudes (ΔF/F_0_) to photoreleased InsP_3_ in InsP_3_R3^+/−^ (grey box, open dots) and InsP_3_R3^−/−^ cells (black box, black dots). All cells responded at least once to a chemostimulus (HMW or E1050). Mann-Whitney test, ***P < 0.001. (**h**) Percentage of InsP_3_R3^+/−^ and InsP_3_R3^−/−^ cells responding to HMW or E1050. HMW: 9.28% (48/517 cells, InsP_3_R3^+/−^) and 13.79% (40/290 cells, InsP_3_R3^−/−^). E1050: 10.24% (38/371 cells, InsP_3_R3^+/−^) and 12.36% (32/259 cells, InsP_3_R3^−/−^). **(i)** Ca^2+^ peak amplitudes (ΔF/F_0_) of cells that were stimulated successively with a given chemostimulus (HMW or E1050), a UV stimulus, and a second chemostimulus. Note that UV-evoked Ca^2+^ responses were absent in InsP_3_R3^−/−^ cells. Kruskal-Wallis test, P < 0.0001; Mann-Whitney test, **P < 0.01; ***P < 0.001. Cell numbers indicated above each bar.

InsP_3_R3 immunoreactivity has been reported in rat VSNs^28^ and transcripts of all three InsP_3_R isoforms were identified in whole mouse VNO tissue^53^. To obtain independent evidence for the presence and function of InsP_3_Rs in mouse VSNs, we investigated the expression of InsP_3_R3 in VNO cryosections of adult WT mice using specific anti-InsP_3_R3 antibodies. We observed InsP_3_R3 immunoreactivity in the entire vomeronasal neuroepithelium, especially in VSN somata, dendrites and possibly in dendritic endings, whereas immunoreactivity was weaker in supporting cell somata and non-sensory parts of the VNO (Fig. 3a). Importantly, this labeling was absent in InsP_3_R3^−/−^ VNO sections, confirming antibody specificity (Fig. 3a). Next, we performed RT-PCR on cDNA libraries prepared from single-cell RNA. We dissociated VNOs from OMP-GFP mice^54^ to obtain single, isolated and fluorescent VSNs that were individually collected using a microcapillary pipette. In OMP-GFP mice, GFP serves as a marker for mature (olfactory marker protein-expressing) VSNs. We picked 10 GFP+ cells and 2 GFP– cells. We prepared RNA from each cell, generated single-cell cDNA libraries^10, 44^, and assessed for gene expression by PCR with gene-specific primers. We amplified *Omp* PCR products in 8/10 GFP+ cells (Fig. 3b), indicative of a 80% success rate for this method. We further screened for *Itpr3* gene expression in these 8 GFP+ cells and obtained *Itpr3* PCR products in 4 cell samples (Fig. 3c), demonstrating that InsP_3_R3 is indeed expressed in at least a fraction of VSNs. Full-length gels are presented in Supplementary Fig. S1. We found no detectable expression of *Itpr1* (InsP_3_R1) or *Itpr2* (InsP_3_R2) genes in all samples, except for one of the GFP− control cells that was positive for *Itpr2* (not shown). For comparison, we also sampled 18 cells from InsP_3_R3^−/−^ VNO (which are GFP−), 8 of which were positive for the *Omp* PCR (Fig. 3b). We did not amplify *Itpr3* PCR products from any of these cells (Fig. 3c). We found *Itpr2* expression in 5 of the cells that were negative for *Omp* (not shown). These results indicate that InsP_3_R3 is the predominant isoform expressed in VSNs, whereas InsP_3_R1 and InsP_3_R2 are not co-expressed in these cells. InsP_3_R2 is likely expressed by supporting and other non-sensory cell types. There was no evidence for upregulation of *Itpr1* or *Itpr2* in InsP_3_R3^−/−^ VSNs.

### Photolysis of caged InsP_3_ shows that InsP_3_R3 is required for Ca^2+^ release

To determine whether InsP_3_ is capable to evoke Ca^2+^ elevations in VSNs and, if so, whether this signal would require InsP_3_R3, we performed flash photolysis experiments with caged InsP_3_ in InsP_3_R3^+/−^ and InsP_3_R3^−/−^ VSNs (Fig. 3d–h). Freshly dissociated VSNs were co-loaded with a photoactivatable and membrane-permeant caged InsP_3_ propionyloxymethyl ester^55^ as well as the Ca^2+^ indicator fluo-4 (see Methods for details). We used confocal laser-scanning microscopy to monitor changes in cytosolic Ca^2+^ and to locally deliver ultraviolet light (UV, 355 nm) stimulation which, in turn, caused photoliberation of caged InsP_3_ and thereby released the active InsP_3_ molecule. Cells exhibiting Ca^2+^ transients in response to either E1050 or HMW were identified as functional VSNs and the regions circumscribed by each cell were outlined and stimulated with UV light (Fig. 3d). A typical UV stimulation for a single cell with a diameter of 10 µm consisted of 10 individual scans with a pixel dwell time of 1.54 µs, resulting in a total UV exposure time of 1.76 ms. With these conditions, InsP_3_R3^+/−^ VSNs showed striking Ca^2+^ transients in response to UV stimulation (Fig. 3d and e) demonstrating that InsP_3_ is indeed capable to induce increases of cytoplasmic Ca^2+^ in these cells. Consistent with previous studies using InsP_3_ photorelease in other cell types^56^, uncaging efficiency in InsP_3_R3^+/−^ VSNs was between 30% and 40%: 14/38 (37%) of E1050-activated cells and 15/48 (31%) of HMW-activated cells showed Ca^2+^ transients after UV exposure (Fig. 3f and g). By contrast, InsP_3_R3^−/−^ VSNs, which showed normal response rates for HMW and E1050 (Fig. 3h), were largely insensitive to InsP_3_ photorelease (Fig. 3d,e,g and i), indicating that InsP_3_R3 is required for InsP_3_-induced Ca^2+^ release in these VSNs. For example, from a total of 32 cells responding to E1050 and 43 additional cells responding to HMW, we observed only a single cell in each group that could be activated by UV light (Fig. 3f and g). Furthermore, when we analyzed only those cells (n = 16 InsP_3_R3^+/−^ VSNs and n = 13 InsP_3_R3^−/−^ VSNs, respectively) that were stimulated successively with a chemostimulus (HMW or E1050), UV light, and a second chemostimulus (Fig. 3i), UV-evoked responses were completely absent in InsP_3_R3^−/−^ versus InsP_3_R3^+/−^ VSNs (Mann-Whitney, P < 0.001). Together, these results show that photolysis of InsP_3_ can induce transient Ca^2+^ elevations in VSNs heterozygous for the InsP_3_R3 mutation but that these responses are absent or strongly reduced in InsP_3_R3-deficient VSNs. Therefore, InsP_3_R3 is the predominant isoform mediating InsP_3_-evoked Ca^2+^ release in these sensory neurons.

### Stimulus-induced Ca^2+^ signaling in InsP_3_R3-deficient VSNs

**Figure 4:**
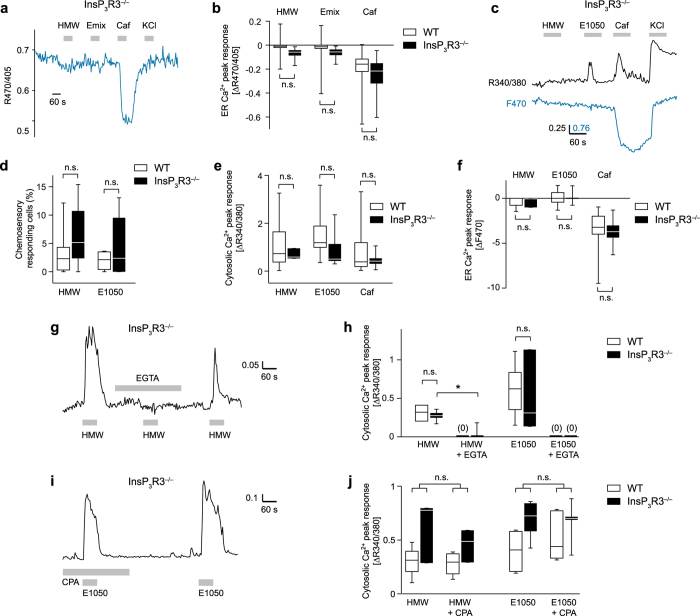
Ligand-evoked Ca^2+^ signals in InsP_3_R3^−/−^ VSNs. (**a**) F_470/405_ ratio of an erGAP2-expressing InsP_3_R3^−/−^ VSN stimulated with HMW (1:300), E mix, caffeine (50 mM) and high K^+^ (100 mM). (**b**) [Ca^2+^]_ER_ peak amplitudes in WT and InsP_3_R3^−/−^ VSNs with erGAP2 imaging (n = 368 and 67 cells, respectively. n.s., not significant; Mann-Whitney test, P = 0.103, caffeine; Kruskal-Wallis test, P = 0.48; HMW and E mix). (**c**) Simultaneous erGAP2 and fura-2 Ca^2+^ imaging of an InsP_3_R3^−/−^ VSN stimulated with HMW (1:300), E1050 (100 µM), caffeine, and high K^+^. (**d**) Percentage of activated cells responding to either HMW or E1050 (fura-2 imaging) in WT (n = 599) or InsP_3_R3^−/−^ (n = 171) VSNs. 15 and 6 cells responded to E1050 and 8 and 7 cells responded to HMW for WT (n = 8) and InsP_3_R3^−/−^ VSNs (n = 6), respectively. n.s. not significant, Mann-Whitney test, P = 0.27 (HMW) and 0.74 (E1050). (**e**) [Ca^2+^]_ER_ peak amplitudes of the cells indicated in (**d**). n.s., not significant, Mann-Whitney test, P = 0.91 (HMW) and 0.12 (E1050). (**f**) [Ca^2+^]_ER_ changes measured with erGAP2 at 470 nm on the same cells. n.s., not significant, Mann-Whitney test, P = 0.83 (caffeine), P = 0.3 (HMW), p = 0.99 (E1050). (**g**) Fura-2-imaged InsP_3_R3^−/−^ VSN activated by HMW. Response to HMW is abolished after removal of extracellular Ca^2+^. (**h**) Analysis of [Ca^2+^]_C_ peak amplitudes in WT and InsP_3_R3^−/−^ VSNs to HMW and E1050. n.s., not significant, Mann-Whitney test, P = 0.47, HMW (n = 3 and 8); P = 0.76, E1050 (n = 8 and 3); HMW vs. EGTA-HMW: *P = 8.3 × 10^−4^ (InsP_3_R3^−/−^). (**i**) E1050-evoked [Ca^2+^]_C_ responses of an InsP_3_R3^−/−^ VSN in presence or absence of CPA. (**j**) [Ca^2+^]_C_ peak amplitudes of WT and InsP_3_R3^−/−^ VSNs elicited by HMW (n = 4 and 3) and E1050 (n = 7 and 5). n.s., not significant, Wilcoxon signed-rank test, HMW vs. CPA-HMW: P = 0.58 (WT) and P = 0.42 (InsP_3_R3^−/−^); E1050 vs. CPA-E1050: P = 0.08 (WT) and P = 0.78 (InsP_3_R3^−/−^).

We performed a systematic analysis of stimulus-evoked Ca^2+^ responses in InsP_3_R3^−/−^ VSNs. We first monitored Ca^2+^ signals in the ER lumen of InsP_3_R3^−/−^ VSNs using ratiometric erGAP2 imaging. We analysed [Ca^2+^]_ER_ in response to caffeine, HMW or E mix in 67 dissociated VSNs expressing erGAP2 via HSV-1 infection. Caffeine induced strong reductions of F_470_/F_405_ ratio, consistent with a decrease of [Ca^2+^]_ER_ (Fig. 4a). Average values of these responses (−0.193) were not significantly different from WT VSNs (Mann-Whitney test, P = 0.103; Fig. 4b). Thus, ER Ca^2+^ loading and caffeine-induced Ca^2+^ release seem to be intact in these InsP_3_R3^−/−^ cells. E mix and HMW induced modest changes in F_470_/F_405_ ratio (−0.0126 and −0.015 a.u., respectively), not significantly different from WT VSNs (Kruskal-Wallis test, P = 0.48; Fig. 4b).

Simultaneous [Ca^2+^]_ER_ and [Ca^2+^]_C_ measurements using erGAP2 and fura-2 imaging in InsP_3_R3^−/−^ cells (Fig. 4c) produced comparable results: we imaged 171 erGAP2-expressing cells loaded with fura-2 and observed responses in 6 cells to HMW and in 7 cells to E1050 (5 separate experiments). The cell activation rate per experiment for both stimuli ranged between 4.6–6.5%, but was not significantly different from WT controls (2–3.2%; Mann-Whitney test, P = 0.27–0.74; Fig. 4d). The average amplitude of [Ca^2+^]_C_ increases in response to HMW and E1050 (0.35–0.41 ratio values) tended to be slightly lower in InsP_3_R3^−/−^ vs. WT cells (0.56–0.75), but this difference was not statistically significant (Mann-Whitney test, P = 0.91–0.12; Fig. 4e). InsP_3_R3^−/−^ cells showed [Ca^2+^]_C_ increases in response to caffeine similar to those observed in WT cells (0.24–0.4; Mann-Whitney test, P = 0.83), indicating that increases in [Ca^2+^]_C_ are not different in InsP_3_R3^−/−^ cells (Fig. 4e). These cells showed large [Ca^2+^]_ER_ (F_470_) reductions for caffeine (−3.76 a.u.), but not for E1050 (+0.09 a.u.) and HMW (−0.33 a.u.) stimuli (Fig. 4f). In all cases, average changes in F_470_ did not differ significantly in InsP_3_R3^−/−^ vs. WT cells (Mann-Whitney test, P = 0.3–0.99; Fig. 4f) indicating that genetic ablation of InsP_3_R3 has little or no effect on [Ca^2+^]_ER_ following stimulation with HMW and E1050. Removal of extracellular Ca^2+^ also abolished HMW and E1050-induced [Ca^2+^]_C_ increases (Fig. 4g) in a similar way for both InsP_3_R3^−/−^ and WT cells (Fig. 4h). Similarly, ER Ca^2+^ depletion with CPA had no effect on [Ca^2+^]_C_ amplitudes induced by HMW and E1050 (Wilcoxon signed-rank test, HMW: P = 0.42, n = 3; E1050: P = 0.79, n = 5) in InsP_3_R3^−/−^ and WT cells (Fig. 4i and j). Although not significant, [Ca^2+^]_C_ amplitudes tended to be somewhat higher in InsP_3_R3^−/−^ VSNs. Interestingly, this tendency is reversed in erGAP2-infected cells after 24 h (Fig. 4e). The reasons for this are not known but argue further against a major contribution of InsP_3_R3-mediated Ca^2+^ release to the cytosolic signal under these conditions. We also note that the number of responding cells cannot be compared directly in freshly dissociated versus erGAP2-infected cells. Together, these results indicate that InsP_3_R3 and Ca^2+^ release from the intracellular stores are not critically required for ligand-evoked cytosolic Ca^2+^ transients and sensory activation of mouse VSNs.

### InsP_3_R3 is not required for activation of VSN sensory currents

**Figure 5:**
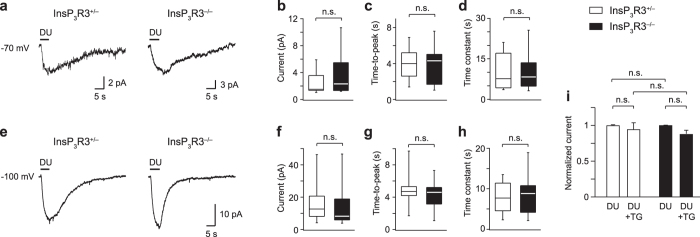
Urine-evoked VSN sensory currents do not require InsP_3_R3. (**a**) Diluted urine (DU, 1:100) elicited sensory currents in both InsP_3_R3^+/−^ (left) and InsP_3_R3^−/−^ (right) VSNs. Holding potential, −70 mV. (**b**) Analysis of peak amplitudes of InsP_3_R3^+/−^ (n = 13) vs. InsP_3_R3^−/−^ (n = 19) VSNs. (**c**) Analysis of time-to-peak values of InsP_3_R3^+/−^ (n = 13) vs. InsP_3_R3^−/−^ (n = 18) VSNs. (**d**) Comparison of decay time constants (τ) of DU-evoked currents (n = 11 and 17, respectively). (**e**) DU-evoked currents in InsP_3_R3^+/−^ vs. InsP_3_R3^−/−^ VSNs recorded at a holding potential of −100 mV (K^+^ reversal potential). **(f)** Analysis of peak amplitudes at −100 mV (n = 23 and 25, respectively). (**g**) Time-to-peak values for all DU responses at −100 mV (n = 23 and 24, respectively). (**h**) Decay time constants (τ) of DU-evoked currents at −100 mV. (**i**) A 5-min preincubation with thapsigargin (TG, 1–5 µM) has no effect on the amplitude of DU-evoked currents (holding potential, −100 mV) in InsP_3_R3^+/−^ (n = 9) or InsP_3_R3^−/−^ (n = 10) VSNs. Median values and interquartile ranges are shown in box plots.

To strengthen our results obtained with Ca^2+^ imaging, we carried out whole-cell patch-clamp recordings in voltage-clamped VSNs^5, 57^. These experiments used acute VNO tissue slices^52, 58, 59^ in which the cellular VSN architecture is preserved, enabling recordings from optically identified VSNs located in apical or basal layers of the sensory epithelium. VSN sensory currents depend, in full or in part, on the activation of Trpc2 cation channels^3–5^. Trpc2 can form a protein-protein interaction complex with InsP_3_R3 and it has been proposed that this interaction contributes to the electrical response of VSNs to chemostimulation^28, 29^. If so, activation of VSN sensory currents should be severely disrupted in InsP_3_R3^−/−^ mice. To assess this, we recorded sensory currents in InsP_3_R3^+/−^ vs. InsP_3_R3^−/−^ VSNs (Fig. 5). Cells were initially stimulated with a mixture of diluted urine from male and female mice (DU, 1:100), a rich source of natural pheromones. This stimulus induced small but reliable inward currents in 19/51 (37%) of InsP_3_R3^+/−^ VSNs and in 20/48 (42%) of InsP_3_R3^−/−^ VSNs, consistent with previous reports of WT VSNs^33, 35, 38^. The properties of urine-evoked sensory currents were analysed in more detail using 5-s urine applications, a holding potential of −70 mV, and a KCl-based intracellular solution (Fig. 5a–d). Under these conditions, we found no significant difference in peak amplitude, time-to-peak, or decay time constants between the two genotypes (Fig. 5a–d and Table 1).

**Table 1:** Properties of sensory currents recorded under voltage-clamp in InsP_3_R3^+/−^ and InsP_3_R3^−/−^ VSNs as indicated in the table. Number of independent experiments (n) is shown in parentheses.

Ligand	Holding potential	InsP_3_R3^+/−^	InsP_3_R3^−/−^	P – value
**Amplitude (pA)**				
DU	−70 mV	2.42 ± 0.44 (13)	3.56 ± 0.66 (19)	0.21
DU	−100 mV	15 ± 2 (23)	13.2 ± 2.1 (25)	0.55
E1050	−70 mV	538 ± 72 (9)	644 ± 133 (6)	0.49
**Time-to-peak (s)**				
DU	−70 mV	4.02 ± 0.45 (13)	3.73 ± 0.48 (18)	0.68
DU	−100 mV	4.9 ± 0.3 (23)	4.3 ± 0.3 (24)	0.22
E1050	−70 mV	5.3 ± 0.6 (9)	5.9 ± 2.3 (5)	0.79
**Time constant (s)**				
DU	−70 mV	10.3 ± 1.9 (11)	9.5 ± 1.4 (17)	0.67
DU	−100 mV	8 ± 0.7 (23)	8.8 ± 1 (23)	0.52
E1050	−70 mV	2.4 ± 0.3 (9)	2.1 ± 0.4 (5)	0.64
**Thapsigargin (normalized response)**				
DU	−100 mV	0.95 (9)		0.68
DU	−100 mV		0.88 (10)	0.31

Ca^2+^-activated K^+^ channels can contribute to urine-evoked currents in VSNs^38^. To determine whether large K^+^ currents would mask the contribution of InsP_3_R3 at resting membrane potential, we recorded urine-evoked currents at a holding potential of −100 mV, close to the K^+^ reversal potential (Fig. 5e–h). This resulted in considerably larger peak amplitudes of urine-evoked currents, but again there was no significant difference between the two genotypes in peak amplitude, time-to-peak, or decay time constant (Fig. 5e–h and Table 1). Furthermore, urine-evoked sensory currents (holding potential, −100 mV) were fully preserved when the tissue was pretreated for 5 min with the SERCA inhibitor thapsigargin (1–5 µM) to deplete intracellular Ca^2+^ stores irreversibly, and there was no significant difference between InsP_3_R3^+/−^ vs. InsP_3_R3^−/−^ VSNs in their peak amplitudes (Fig. 5i and Table 1). These results indicate that Ca^2+^ release from intracellular stores is not essential for urine-evoked VSN sensory currents.

**Figure 6:**
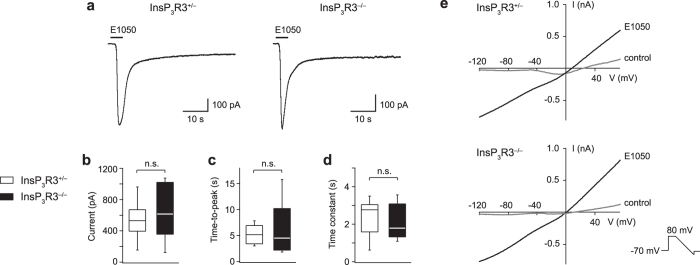
No requirement of InsP_3_R3 for β-estradiol 3,17-disulfate (E1050)-evoked VSN sensory currents. (**a**) E1050 (10 nM) induced large inward currents in both InsP_3_R3^+/−^ and InsP_3_R3^−/−^ VSNs. Holding potential, −70 mV. (**b**) Analysis of E1050-evoked peak amplitudes of InsP_3_R3^+/−^ (n = 9) and InsP_3_R3^−/−^ (n = 6) VSNs. (**c**) Time-to-peak values of E1050-evoked current of InsP_3_R3^+/−^ (n = 9) and InsP_3_R3^−/−^ (n = 5) VSNs. (**d**) Decay time constants (τ, single exponential fits) of E1050-evoked currents responses of InsP_3_R3^+/−^ (n = 9) and InsP_3_R3^−/−^ (n = 5) VSNs. **(e**) Examples of current-voltage (I-V) curves, measured with voltage ramps (duration: 60 ms, slope: −3.3 mV/ms) elicited at the peak of E1050-evoked currents in VSNs of the two different genotypes. Control I-V curves obtained without E1050 stimulation are shown for comparison. Median values and interquartile ranges are shown in box plots.

We also analysed sensory currents evoked by the sulfated steroid E1050 (Fig. 6 and Table 1). This stimulus routinely evoked large inward currents with peak amplitudes of several hundred picoamperes, even at a concentration of only 10 nM (Fig. 6a and b). However, we found no significant difference in peak amplitude, time-to-peak and decay time constant between the two genotypes (Fig. 6b–d and Table 1). Furthermore, current-voltage curves obtained at the peak of E1050-evoked responses (CsCl-based intracellular solution) where very similar between the two genotypes and showed a reversal potential close to 0 mV (InsP_3_R3^+/−^: V_rev_ = 4.3 ± 2 mV; InsP_3_R3^−/−^: 1.4 ± 1.2 mV, n = 3 each) (Fig. 6e). Therefore, InsP_3_R3 is not essential for the activation of VSN sensory currents.

### Normal action potential responses in InsP_3_R3^−/−^ VSNs

**Figure 7:**
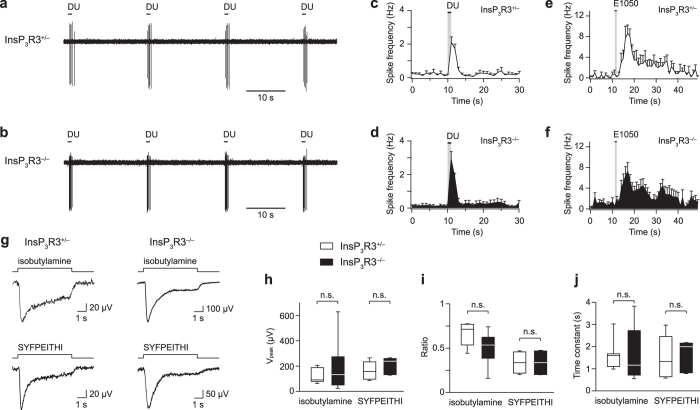
No requirement of InsP_3_R3 for VSN action potential responses or Ca^2+^-CaM-dependent adaptation. (**a**,**b**) Repeated DU pulses (1 s duration, 20 s interval) evoke action potential responses in InsP_3_R3^+/−^ and InsP_3_R3^−/−^ VSNs. (**c**,**d**) Group data showing PSTH analyses of DU-evoked action potential responses from InsP_3_R3^+/−^ (n = 16) and InsP_3_R3^−/−^ (n = 18) VSNs. (**e**,**f**) Group data showing PSTH analyses of action potential responses to E1050 (10 nM) from InsP_3_R3^+/−^ (n = 5) and InsP_3_R3^−/−^ (n = 7) VSNs. (**g**) Examples of VNO field potential responses to 6-s pulses of isobutylamine (0.1 µM) or SYFPEITHI (1 nM) in InsP_3_R3^+/−^ and InsP_3_R3^−/−^ mice. (**h–j**) Analyses of field potential peak responses (unpaired t-test: isobutylamine: t(12) = 0.76, P = 0.46; SYFPEITHI: t(5) = 0.76, P = 0.48) (**h**), ratio between plateau and peak (unpaired t-test: isobutylamine: t(12) = 2.07, P = 0.06; SYFPEITHI: t(5) = 0.09, P = 0.93) (**i**), and time constant of adaptation onset (single exponential fit, unpaired t-test: isobutylamine: t(8) = 1.08, P = 0.31; SYFPEITHI: t(5) = 0.14, P = 0.89) (**j**) for the two ligands and the two genotypes as indicated. Results are based on 4–8 independent recordings from 3–5 mice for each ligand and genotype.

Our results of Figs 5 and 6 were further supported by extracellular loose-patch recordings from VSNs in tissue slices measuring stimulus-evoked action potential sequences (Fig. 7a–f). These represent VSN output signals that are ultimately transmitted to the olfactory forebrain. Using 1-s pulses of diluted urine as stimulus, action potential responses could be readily evoked and repeated multiple times in both InsP_3_R3^+/−^ and InsP_3_R3^−/−^ VSNs (Fig. 7a and b). Post-stimulus time histograms (PSTHs) obtained from group data of such recordings revealed no significant difference between the two genotypes (P = 0.07–0.97) (Fig. 7c and d). The same basic result was also observed for action potential sequences in response to E1050 (10 nM, P = 0.06–1) (Fig. 7e and f).

### InsP_3_R3 is not required for Ca^2+^-calmodulin-dependent VNO adaptation

The N-terminus of Trpc2 binds CaM in a Ca^2+^-dependent manner^60^. We showed previously that Ca^2+^-CaM feedback mediates sensory adaptation and inhibits DAG-activated Trpc2 currents in VSNs^32^. For other TRPC channels such as Trpc3, an activation model has been proposed in which an InsP_3_R binds to a site that partially overlaps with the Ca^2+^-CaM site, thereby displacing CaM and thus causing channel activation^61^. We tested whether activation and Ca^2+^-CaM-dependent VSN adaptation is altered in the absence of InsP_3_R3 by performing extracellular field potential recordings from the surface of the sensory epithelium using an intact VNO wholemount preparation^32, 52, 58, 62, 63^ (Fig. 7g–j). We recorded isobutylamine-evoked potentials (0.1 µM), which depend on type 1 vomeronasal receptors^62^, and potentials to SYFPEITHI (1 nM), a major histocompatibility complex (MHC) binding peptide^58^. SYFPEITHI-evoked responses require type 2 vomeronasal receptors and the G protein Gαo^47, 59^ but persist in Trpc2-deficient mice^64^. We found that both ligands evoked robust, phasic-tonic field potentials in InsP_3_R3^+/−^ and InsP_3_R3^−/−^ VNO that underwent time-dependent desensitization with prolonged stimulation (Fig. 7g). We analysed field potential peak amplitudes, the ratio between plateau and peak as a measure of the extent of adaptation, and the adaptation onset time constant^32^ (Fig. 7h–j). Despite a slight trend for lower plateau-peak ratios with isobutylamine, there was no significant difference between the two genotypes for both stimuli in any of these parameters (unpaired t-test: P = 0.09–0.93). These results indicate that InsP_3_R3 is neither required for the activation of ligand-evoked field potentials in the VNO nor for their Ca^2+^-CaM-dependent desensitization.

## Discussion

The role of the second messenger InsP_3_ in vertebrate olfaction has been discussed controversially for almost 30 years, but no gene deletion studies have been performed to address this critical problem in any of the olfactory subsystems for any vertebrate species. To overcome this limitation, we undertook a series of investigations employing InsP_3_R3-deficient mice. We focused on sensory neurons of the VNO because these have been shown previously to express InsP_3_R3 but not InsP_3_R1 or InsP_3_R2^28^. In agreement with these results, our knockout-controlled immunolabeling and single-cell RT-PCR experiments confirmed the presence of InsP_3_R3 RNA and protein in mouse VSNs but found no evidence for other InsP_3_Rs in these cells. We used a confocal, laser-scanning-controlled approach to photorelease InsP_3_ and simultaneously monitor cytosolic Ca^2+^ in isolated VSNs. These experiments demonstrated for the first time that InsP_3_ evokes transient, intracellular Ca^2+^ rises in VSNs and that InsP_3_R3 is functionally required for this effect, providing a robust foundation for investigating the role of InsP_3_R3 in sensory activation of the mouse VNO. We applied a wide range of vomeronasal chemostimuli and used multiple recording techniques and VNO preparations to address a potential role of InsP_3_R3. Our approach included Ca^2+^ imaging as well as voltage-clamp, loose-patch, and field-potential recordings in isolated VSNs, acute VNO tissue slices, and a VNO wholemount preparation. All of these experiments led to the same basic result, namely that InsP_3_R3 is dispensable for sensory activation of the VNO and, therefore, does not contribute crucially to the primary, chemoelectrical transduction process of VSNs. We also demonstrated that InsP_3_R3 is not required for Ca^2+^-CaM-dependent VSN adaptation. As a whole, these experiments call for a revision of current schemes suggesting that InsP_3_R3 has a critical function in VSN sensory activation. We note that our experiments do not rule out a potential role for InsP_3_ signaling or InsP_3_R3 function in secondary or modulatory signaling events of mammalian VSNs, nor do they rule out distinct VSN signaling mechanisms in lower vertebrates^65^. We also cannot exclude that primary signaling of noncanonical VSNs, such as those expressing formyl peptide receptor^66^ or odorant receptor genes^67^, would require InsP_3_R3. Future experiments will be needed to address these questions.

Our results have important implications for the gating mechanism of Trpc2 cation channels and, hence, for the TRP channel field in general. Most importantly, our results rule out any gating model in which a physical link of InsP_3_R3 with Trpc2, either directly or indirectly, mediates the opening of the Trpc2 channel in VSNs. Consequently, these results also rule out any model in which the displacement of inhibitory Ca^2+^-CaM by InsP_3_R3 causes Trpc2 activation. The present results are fully consistent with and strengthen further our previous findings that Trpc2 is a DAG-activated, Ca^2+^-permeable cation channel that can be inhibited by Ca^2+^-CaM and does neither require InsP_3_ nor Ca^2+^ release from intracellular stores for its activation in VSNs^5, 7, 18, 32^. These experiments leave the activation of Trpc2 by DAG, possibly in conjunction with PIP_2_, as the most plausible VSN primary transduction mechanism. It remains to be seen whether this model applies also to other Trpc2-expressing cells outside the VNO where cells likely exhibit different cellular architecture or express other Trpc2 splice variants and interacting proteins. It has been suggested that modes of Trpc2 activation are cell-specific and require different interactions and activators in different cell types^14^. Our results should also be of general interest to the activation models of other DAG-sensitive TRP channels such as TRPC3, TRPC6, and TRPC7^68, 69^.

There is considerable evidence that Ca^2+^-activated chloride channels in VSNs, for which TMEM16A/anoctamin1 is an essential component^37^, serve to amplify the sensory response and cause further VSN depolarization because of elevated intracellular Cl^−^ concentrations^33, 35, 70, 71^. In dorsal root ganglia, activation of anoctamin1 by localized Ca^2+^ signals requires coupling with the type 1 InsP_3_ receptor^72^. Similarly, it has been proposed that activation of VSN chloride channels is triggered by Ca^2+^ release from intracellular stores^35^. Our electrophysiological recordings in InsP_3_R3-deficient VSNs found no evidence for a significant reduction of the size of sensory responses. This argues strongly that chloride channel activation in VSN sensory responses is not mediated by InsP_3_R3-dependent Ca^2+^ release.

Several interesting results emerge from our study with respect to the role of sulfated steroids as VSN ligands. Initially, these molecules were tested at relatively high concentrations, at 100 or 200 µM, using extracellular spike recordings^46^ or Ca^2+^ imaging^48^, but the primary sensory currents evoked by these ligands have not been analyzed previously. Using patch-clamp recordings under voltage-clamp, we found that VSNs in VNO slices generate surprisingly large currents at only 10 nM of E1050 (Fig. 6) and also evoke action potentials at this concentration (Fig. 7). These low thresholds are supported by a previous study identifying V1rj receptors that selectively respond to sulfated steroids (including E1050) at 10 nM^73^. Our response rates for E1050 are also consistent with a report that found approximately 0.2–5.5% of the VSNs to be responsive to individual sulfated steroid components including E1050^74^. We cannot yet determine whether the differences in amplitude or response kinetics reflect specific differences in the underlying mechanisms of the sensory currents but, importantly, there was no obvious effect of the InsP_3_R3 deletion on responses to E1050, neither at low nor at high stimulus conditions.

Our experiments demonstrate for the first time the suitability of a novel, genetically-encoded Ca^2+^ sensor protein, erGAP2, for monitoring Ca^2+^ dynamics in the ER of mammalian VSNs. erGAP2 is a ratiometric low-affinity Ca^2+^ sensor of the GFP-aequorin protein family that has been optimized for measurements in high Ca^2+^ concentration environments and that can be targeted to intracellular organelles^42, 43^. These experiments, together with pharmacological manipulations of store-dependent Ca^2+^ mobilization, provided clear evidence that Ca^2+^ release from the ER does not play a critical role in the primary transduction mechanism of mouse VSNs, results that are fully consistent with previous conclusions^5^.

In summary, the present study provides important new insights into the primary mechanisms underlying chemoelectrical signal transduction of the mammalian VNO and are necessary for advancing our understanding of Trpc2 activation. Our data show conclusively that InsP_3_R3 is not required for these functions. These results advance substantially our understanding of the molecular mechanisms mediating sensory activation of the mammalian VNO.

## Materials and Methods

### Mice

All animal protocols complied with the ethical guidelines for the care and use of laboratory animals issued by the German Government and were approved by the Animal Welfare Committee of Saarland University School of Medicine. All methods were carried out in accordance with the relevant guidelines and regulations. Mice were housed in ventilated cages under a 12:12 hour light/dark cycle with food and water available *ad libitum*. Generation of mice that carry a knockout mutation in the *Itpr3* locus and thus lack InsP_3_R3 (InsP_3_R3^−/−^) has been described^40, 41^. This strain was intercrossed with C57BL/6 mice for at least twelve times before use^41^. C57BL/6 mice (denoted as wild-type, WT) or InsP_3_R3^+/−^ littermates were used as reference mice. Some experiments were performed on OMP-GFP mice (heterozygous for the mutation) in which all cells expressing olfactory marker protein (OMP) are genetically labeled and show robust GFP fluorescence^54^. All experiments were performed on adult, 6–18 weeks old mice (both sexes).

### Live-cell Ca^2+^ imaging

Ca^2+^ imaging of freshly dissociated VSNs was performed as described^45, 47, 51^. VNO epithelium was detached from the cartilage and minced in PBS at 4 °C. The tissue was incubated (20 min at 37 °C) in PBS supplemented with papain (0.22 U/ml) and DNase I (10 U/ml; Fermentas), gently extruded in DMEM (Invitrogen) supplemented with 10% FBS, and centrifuged at 100 × g (5 min). Dissociated cells were plated on coverslips previously coated with concanavalin-A type V (0.5 mg/ml, overnight at 4 °C; Sigma). Cells were used immediately for fura-2 imaging after loading them with fura-2/AM (5 µM; Invitrogen) for 60 min, or they were infected with HSV-1 encoding erGAP2 virus and incubated at 37 °C in FBS-supplemented DMEM medium for 24 h before imaging^44^. Coverslips containing VSNs were placed in a laminar-flow chamber (Warner Instruments) and constantly perfused at 22 °C with extracellular solution Hank’s balanced salt solution (HBSS, Invitrogen) supplemented with 10 mM Hepes (2-[4-(2-hydroxyethyl)piperazin-1-yl]ethanesulfonic acid). Cells were alternately epi-illuminated at 405 and 470 nm for erGAP2 imaging, or at 340 and 380 nm for fura-2 imaging, and light emitted above 510 nm was recorded using a C10600-10B Hamamatsu camera installed on an Olympus IX71 microscope. For simultaneous recordings of [Ca^2+^]_ER_ and [Ca^2+^]_C_, erGAP2-expressing cells were incubated for 60 min with fura-2/AM and sequentially excited at 340, 380 and 470 nm. We recorded emitted light at wavelengths >510 nm. The ratio F_340/380_ was used as an index of [Ca^2+^]_C_ and F_470_ as an index of [Ca^2+^]_ER_. Images were acquired at 0.25 Hz and analysed using ImageJ (NIH), including background subtraction, region of interest (ROI) detection and signal analyses. ROIs were selected manually and always included the whole cell body. Peak signals were calculated from the temporal profiles of image ratio/fluorescent values. Results are based on recordings from 3–5 mice for each condition and genotype.

### Chemostimulation

Chemostimuli for Ca^2+^ imaging were prepared fresh daily and diluted in extracellular solution giving the following final concentrations: HMW fraction, 1:300 dilution; sulfated estrogen mix (E mix): E1050 (1,3,5(10)-estratrien-3, 17β-diol disulphate), E1100 (1,3,5(10)-estratrien-3, 17β-diol 3-sulphate), E0893 (1,3,5(10)-estratrien-3, 17α-diol 3-sulphate) and E0588 (17β-dihydroequilin D 3-sodium sulphate), each at 100 µM (Steraloid); E1050, 100 µM; caffeine, 50 mM; KCl, 100 mM. Sulfated estrogens were initially prepared in dimethyl sulfoxide (DMSO) and further diluted in extracellular solution. To obtain HMW fraction, 0.5 ml of fresh urine was collected from adult C57BL/6 males (8–12 weeks old, sexually naïve)^47^ and size-fractionated by centrifugation (14,000 × g for 30 min) using Microcon 10-kDa molecular mass cutoff ultrafiltration columns (Millipore). The centrifugation retentate was washed with 0.5 ml of PBS three times and resuspended in 0.5 ml of PBS. Ca^2+^-free solution was prepared by adding 0.5 mM EGTA (ethylene glycol-bis(β-aminoethyl ether)-N,N,N′,N′-tetraacetic acid) to the extracellular solution. In some experiments, cells were incubated for 20 min at RT in extracellular solution containing 30 µM cyclopiazonic acid (CPA) to deplete intracellular Ca^2+^ stores before application of HMW or E1050.

### Photorelease of InsP_3_

VNO cells were dissociated and plated on coverslips as described above and loaded with 3 μM caged InsP3/PM [D-2,3-O-isopropylidene-6-O-(2-nitro-4,5-dimethoxy)benzyl-myo-inositol 1,4,5-trisphosphate-hexakis (propionoxymethyl)ester; Enzo Life Sciences, Switzerland] mixed with the same volume of Pluronic F127 in DMSO (10%) in Hepes-HBSS buffer. Cells were loaded with caged InsP3/PM for 30 min at room temperature in the dark followed by an additional 30 min incubation of 2.5 μM fluo-4/AM (Invitrogen) and InsP3/PM. Stock solutions were made in DMSO and kept for up to 1 week stored at −20 °C. The final DMSO concentration did not exceed 0.5%. Coverslips containing VSNs were placed in a laminar-flow chamber (Luigs and Neumann) and constantly perfused with extracellular Hepes-buffered solution. We used an upright scanning confocal microscope (Zeiss LSM 880 Indimo) equipped with a standard Argon laser for excitation at wavelength of 488 nm (fluo-4 excitation) and a UV laser (Coherent) emitting 355 nm (InsP3 uncaging). Images were acquired at 0.5 Hz. Emitted fluorescence was collected between 500 and 560 nm. All scanning head settings were kept constant during each experiment. The UV laser light was coupled to the confocal microscope and focused onto the image plane through a 20 × 1.0 NA Plan-Apochromat water immersion objective (Zeiss). The depth of focus was 16 µm which ensured, together with the region of interest (ROI) diameter, illumination of individual cells. Before starting photolysis of caged InsP_3_, UV laser light was optimally focused using 18 µm thick brain tissue sections loaded with Hoechst 33342 (1:10000; ThermoFisher) and the semi-automated correction tool of the Zen software (Zeiss). Photolysis of caged InsP_3_ was achieved by directing UV laser light (1.036 mW) on preselected ROIs (spot diameter ~10 µm) using the Zen software (Zeiss) before reverting back to the visible wavelength laser to resume monitoring of fluo-4 fluorescence. Photorelease of caged InsP_3_ with ROI spot illumination was performed on single cells previously identified to respond to HMW or E1050. In some experiments, we used UV whole-field illumination (ROI area, 425 × 425 µm) to photorelease InsP_3_ in a larger area containing multiple fluo-4 loaded cells, in order to record also from IP_3_R3-deficient cells that did not respond to HMW or E1050 but were potentially sensitive to InsP_3_ (such as non-VSN cell types present in the VNO and expressing IP_3_R1 or IP_3_R2, serving as positive controls). The estimated intracellular InsP_3_ concentration after photolysis was expected to be in the 0.1–5 μM range^56^. Ca^2+^ changes were generally expressed as relative fluorescence changes, i.e. ΔF/F_0_ (F_0_ was the average of the fluorescence values of 5–10 frames before stimulation). Images were acquired at 0.5 Hz and analysed using ImageJ (NIH). Peak Ca^2+^ signals evoked by photoreleased InsP_3_ were calculated from the temporal profiles of ROI values during the first 10–20 s after a UV stimulus.

### Virus production

Virus production, packaging of herpes simplex virus type 1 (HSV-1) vectors and VSN infection was performed as described^44^. This virus-based amplicon delivery system was initially developed to overexpress vomeronasal receptors of the V1r, V2r, and Fpr families in VSNs^44^. To produce viral vectors, a HindIII/EcoRI fragment containing the erGAP2 cDNA was cloned into the herpes simplex virus plasmid pHSVpUC. Packaging and titration of virus particles were performed as reported earlier^75^. VSN cultures were infected with a multiplicity of infection (moi) ranging between 0.01 and 0.1 one day before use.

### Immunohistochemistry

Mice were deeply anesthetized with CO_2_ prior to decapitation and VNOs were removed, fixed for 3 h in 4% paraformaldehyde, equilibrated overnight in PBS containing 30% sucrose, embedded in OCT (Tissue-Tek), and snap-frozen in a dry ice/2-methylbutane bath. Frozen tissue sections (16 µm) were collected on glass slides (Superfrost Plus, Polysciences) and stored at −80 °C until use. Sections were post-fixed 15 min in 4% paraformaldehyde, washed 3 times in PBS (10 min each), incubated in blocking solution (0.5% Triton X-100, 3% horse serum, in PBS) for 3 h, and incubated overnight at 4 °C in blocking solution containing anti-InsP_3_R3 primary antibody (1:500, Millipore). The tissue was then washed 3 times in PBS (10 min each), incubated in AlexaFluor 488 goat anti-rabbit secondary antibody (1:500, Vector) 1 h at room temperature, and in Hoechst (10 µg/ml, Life Technologies) 5 min at RT. Sections were mounted using Vectashield Mounting Medium (Vector). Fluorescent images were acquired on a Nikon 80i microscope.

### Single-cell RT-PCR

Single-cell cDNA libraries were prepared as described^10, 44^. VSNs were collected using a glass capillary (~10 m tip size) in 1 µl of extracellular solution. Single cells were then transferred to a PCR tube containing 1 µl of diethylpyrocarbonate (DEPC)-treated water. As control, 1 µl of extracellular solution containing no cells was collected with the glass capillary. Samples were immediately frozen on dry ice and kept at −80 °C until use. Second rounds of PCR were performed using the following primers: *Omp*: GCACAGTTAGCAGGTTCAGCT and GGTTTGCAGTCCTGGCAGC; *Itpr1*: CGAGGCTGGAAATGAAGGGT and CCACTGAGGGCTGAAACTCC; *Itpr2*: GGCTGCAAAGAGGTGAATGC and GACGCGATGTCATTTCCGTG; *Itpr3*: TGCCATGTCCCTGGTGAGC and GACCTGAAGGAAGGGCAGTG. PCR products were sequenced to exclude unspecific amplification or false positives. Total VNO mRNA was obtained from pooled VNO tissue of C57BL/6 adult mice (both genders) using PureLink RNA Mini Kit (Ambion) according to the manufacturer’s instructions. Traces of genomic DNA were digested by incubation with 30 U of DNase I (Fermentas).

### Electrophysiology

Whole-cell voltage-clamp or loose-patch recordings from optically identified VSNs were performed in acute VNO tissue slices^5, 32, 57^. The recording chamber was perfused at a rate of ~1 ml/min with bicarbonate-buffered oxygenated (95% O_2_/5% CO_2_) extracellular solution (ACSF) containing (in mM) 125 NaCl, 25 NaHCO_3_, 2.5 KCl, 1 MgCl_2_, 2 CaCl_2_, 1.25 NaH_2_PO_4_, 10 glucose; pH, 7.3; 300 mOsm. The intracellular solution contained, in mM: KCl 140, EGTA 1, Hepes 10, Mg-ATP 2, Na-GTP 1, pH, 7.1; 290 mOsm for urine-evoked responses and: CsCl 140, EGTA 1, Hepes 10, Mg-ATP 2, Na-GTP 1, pH, 7.1; 290 mM for some E1050-evoked currents. Recordings were performed at room temperature using an EPC-9 patch clamp amplifier (HEKA Elektronik, Lambrecht, Germany) and Pulse 8.80 software. In voltage-clamp experiments the membrane potential was clamped to −70 mV, if not otherwise noted. For urine-evoked responses, we chose VSNs from both apical and basal layers. For E1050-evoked responses we focused on VSNs located in the apical layer. In some experiments, we elicited voltage ramps (duration, 60 ms; slope of −3.3 mV/ms; from 80 mV to −120 mV) at the peak of sensory currents. Urine samples were freshly collected from mature C57/BL6 mice (either sex) and frozen for up to three months at −80 °C. Chemostimuli were delivered for 1 or 5 s via multi-barrel pipettes using a pressurized perfusion system (Picospritzer II, General Valve Corp.). Extracellular loose-patch recordings were performed as described^52, 58^. Local field potentials from intact VNO (fluid phase) were recorded as described previously^32, 47, 52, 58, 63^.

### Statistics

Student’s *t* test and Mann-Whitney U test were used for measuring the significance of difference between two independent distributions, Wilcoxon signed-rank test was used when comparing two related samples, and Kruskal-Wallis test for three or more independent samples. Analyses were performed using Origin8.6 (OriginLab) or Igor Pro (WaveMetrics) software. Unless otherwise stated, results are presented as means ± SEM. Box-whisker plots show median values, mininimum-maximum outliers, and interquartile (25–75%) ranges.

### Data availability statement

The datasets generated and/or analysed during the current study are available from the corresponding author on reasonable request.

## Electronic supplementary material

Supplementary Figure S1
