# Supplementary material for: Type 3 inositol 1,4,5-trisphosphate receptor is dispensable for sensory activation of the mammalian vomeronasal organ
Source: Sci Rep. 2017 Aug 31;7:10260. doi: 10.1038/s41598-017-09638-8 (PMC5579292; doi:10.1038/s41598-017-09638-8)
Supplement: Supplementary file 1 — Supplementary Figure S1 [file 41598_2017_9638_MOESM1_ESM.pdf]

Supplementary information

Type 3 inositol 1,4,5-trisphosphate receptor is dispensable for sensory activation of the mammalian vomeronasal organ

Pablo Chamero[1,2], Jan Weiss[1], María Teresa Alonso[3], Macarena Rodríguez-Prados[3], Chihiro Hisatsune[4], Katsuhiko Mikoshiba[4], Trese Leinders-Zufall[1] & Frank Zufall[1*]

[1]Center for Integrative Physiology and Molecular Medicine, Saarland University, 66421 Homburg, Germany. [2]Laboratoire de Physiologie de la Reproduction et des Comportements, UMR 0085 INRA-CNRS-IFCE-Université de Tours, Nouzilly, 37380, France. [3]Instituto de Biología y Genética Molecular (IBGM), University of Valladolid and CSIC, 47003 Valladolid, Spain. [4]Laboratory for Developmental Neurobiology, RIKEN Brain Science Institute, Saitama 351-0198, Japan.

*Correspondence and requests for materials should be addressed to F.Z. (email: frank.zufall@uks.eu)

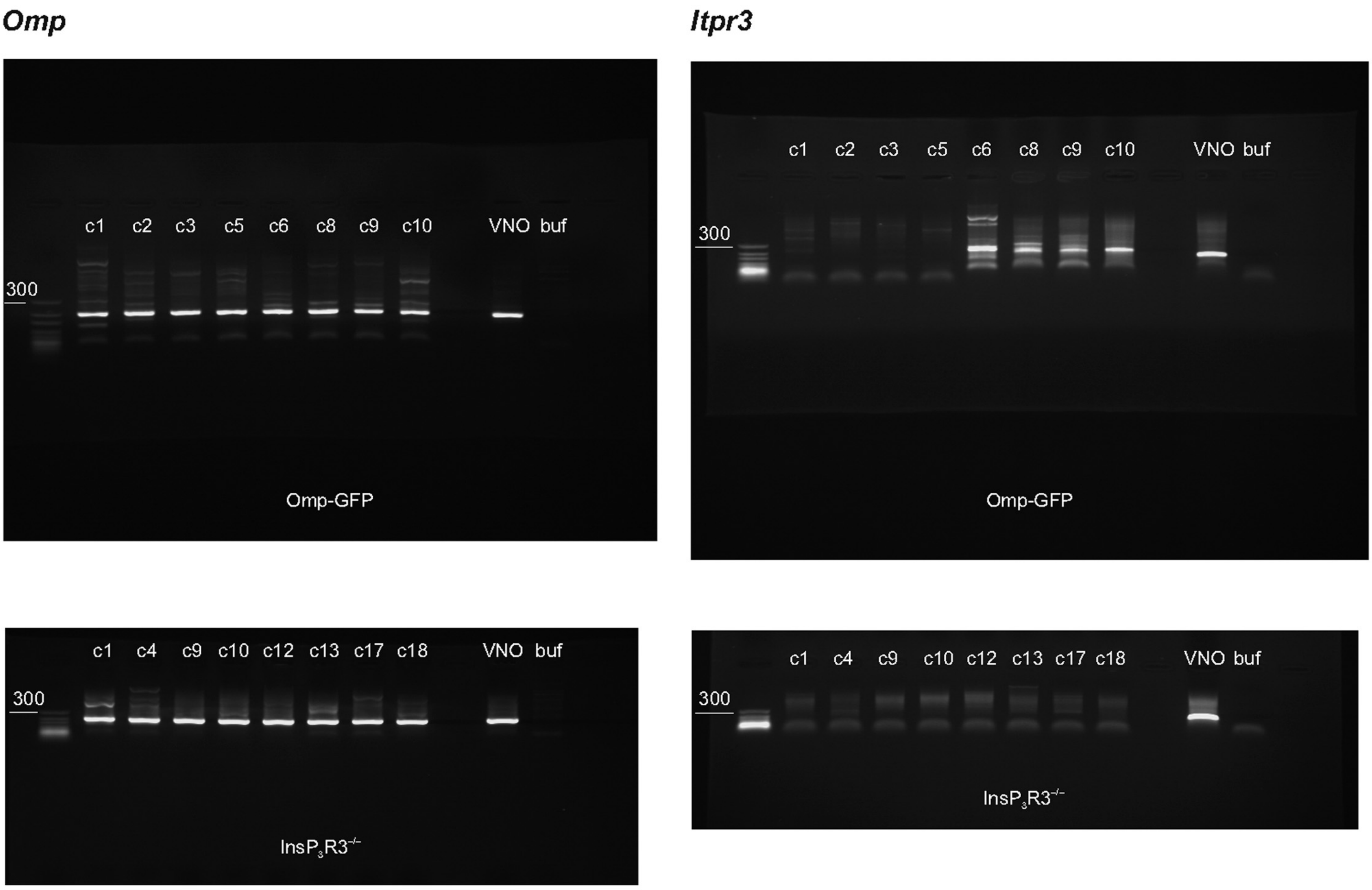

Supplementary Figure S1. Full length gels presented in Fig. 3b and 3c.
